# Effects of intraovarian injection of autologous platelet rich plasma on ovarian reserve and IVF outcome parameters in women with primary ovarian insufficiency

**DOI:** 10.18632/aging.103403

**Published:** 2020-06-05

**Authors:** Yigit Cakiroglu, Ayse Saltik, Aysen Yuceturk, Ozge Karaosmanoglu, Sule Yildirim Kopuk, Richard T. Scott, Bulent Tiras, Emre Seli

**Affiliations:** 1 Acibadem Maslak Hospital Assisted Reproductive Technologies Unit, Istanbul, Turkey; 2 IVI RMA New Jersey, Basking Ridge, NJ 07920, USA; 3 Department of Obstetrics and Gynecology, Thomas Jefferson University, Philadelphia, PA 19107, USA; 4 Acibadem University, Istanbul, Turkey; 5 Department of Obstetrics, Gynecology and Reproductive Sciences, Yale School of Medicine, New Haven, CT 06510, USA

**Keywords:** primary ovarian insufficiency, platelet rich plasma, PRP, in vitro fertilization

## Abstract

We aimed to determine whether intraovarian injection of autologous platelet rich plasma (PRP) improves response to ovarian stimulation and in vitro fertilization (IVF) outcome in women with primary ovarian insufficiency (POI). Women (N=311; age 24-40) diagnosed with POI based on ESHRE criteria underwent intraovarian PRP injection. Markers of ovarian reserve, and IVF outcome parameters were followed. PRP treatment resulted in increased antral follicle count (AFC) and serum antimullerian hormone (AMH), while serum follicle stimulating hormone (FSH) did not change significantly. After PRP injection, 23 women (7.4%) conceived spontaneously, 201 (64.8%) developed antral follicle(s) and attempted IVF, and 87 (27.8%) had no antral follicles and therefore did not receive additional treatment. Among the 201 women who attempted IVF, 82 (26.4% of total) developed embryos; 25 of these women preferred to cryopreserve embryos for transfer at a later stage, while 57 underwent embryo transfer resulting in 13 pregnancies (22.8% per transfer, 4% of total). In total, of the 311 women treated with PRP, 25 (8.0%) achieved livebirth/sustained implantation (spontaneously or after IVF), while another 25 (8.0%) cryopreserved embryos. Our findings suggest that in women with POI, intraovarian injection of autologous PRP might be considered as an alternative experimental treatment option.

## INTRODUCTION

Ovarian aging is a physiological process associated with a decline in the quantity and quality of the oocytes stored within the follicular cohort [1]. Age-related physiological decline in the number of follicles has critical implications for fertility and is an increasingly more prevalent reason for women to seek fertility treatment. Indeed, the number of women undergoing infertility treatment with assisted reproductive technologies (ART) in the United States with a diagnosis of diminished ovarian reserve (DOR) increased from 22,089 in 2010, to 40,883 in 2017 [2, 3]. The extreme form of DOR, primary ovarian insufficiency (POI), affects 1% of reproductive age women, and is characterized by a severe decrease in ovarian reserve prior to 40 years of age, resulting in menopausal serum gonadotropin hormone levels and menstrual irregularity or amenorrhea [4]. The European Society of Reproductive Medicine and Embryology guidelines define POI as the presence of oligomenorrhea-amenorrhea for at least 4 months and serum follicle stimulating hormone (FSH) levels of ≥ 25 IU/ml measured at least twice with a 4-week interval, with an onset before the age of 40 years [5].

Currently, oocyte donation is the only established treatment modality for women with POI [6]. Similar to the trend observed in women diagnosed with DOR, number of women undergoing oocyte donation in the United States increased from 15,504 in 2010 to 21,033 in 2017 [2, 3]. While utilization of oocyte donation continues to increase, a large number of women express having difficulty adjusting to the idea of not having a child that carries their own genetic material. Another impediment to oocyte donation is that a number of countries have limitations regarding the use of donor oocytes due to ethical or religious concerns, compelling women to seek experimental therapies.

At present, there are no effective treatment modalities for women with POI who wish to have children with their own eggs. While a number of experimental strategies have been tested, live births have been difficult to achieve in women with POI [7]. Kawamura et al. have performed Hippo pathway disruption and Akt activation [8]. First, they have removed ovaries from women with POI and cryopreserved ovarian cortical strips. They then fragmentated the ovarian strips to disrupt Hippo signaling, stimulated Akt signaling in-vitro, and grafted the fragments back to the ovaries. In the first study using this approach involving 27 women, they reported a live birth after in vitro fertilization (IVF) and transfer of an embryo. In a follow up study from the same group, among 37 women with POI, 9 achieved follicle growth in auto-grafts, 6 underwent oocyte retrieval, and 4 underwent embryo transfer, resulting in two live births (5.4%) [9]. Experimental treatment modalities have also been used in women with DOR. Herraiz et al. have investigated the effects of autologous stem cell ovarian transplant (ASCOT) on ovarian reserve in poor responders [10]. In this study, bone marrow derived stem cells (BMDSC) were mobilized to peripheral blood by granulocyte colony-stimulating factor (G-CSF) treatment and subsequently collected by apheresis. Cells were delivered into the ovarian artery by an intra-arterial catheter. Seventeen women were treated, resulting in increased number of antral follicles and oocytes, and five pregnancies, three of which were spontaneous. While ASCOT is a complex procedure, their study is encouraging regarding the potential effect of bone marrow or blood-derived substances on follicle development in women with a limited ovarian reserve.

Platelet Rich Plasma (PRP) consists of a high concentration of platelets found in plasma obtained after centrifugation of peripherally collected blood [11]. Platelets carry more than 800 types of protein molecules, cytokines, hormones, and chemoattractants [12]. The activation of platelets induces the release of a variety of biologically active proteins, which stimulate cell proliferation, growth, and differentiation [13]. Biologically active mediators relased by activated platelets include platelet-derived growth factor (PDGF), transforming growth factor-β (TGF-β), fibroblast growth factor (FGF), epidermal growth factor (EGF), insulin-like growth factor-1 (IGF-1), and hepatocyte growth factor (HGF) [14]. PRP induces accelerated angiogenesis and anabolism, inflammation-control, cell migration, differentiation and proliferation [15]. Autologous platelets have been used as a source of proteins for tissue healing and regeneration and have been suggested to promote the development of isolated human primordial and primary follicles to the preantral stage [16].

In this study, we hypothesized that intraovarian injection of autologous PRP would improve ovarian reserve parameters, ovarian response to stimulation, and IVF outcomes in women diagnosed with POI. We treated 311 women previously diagnosed with POI with intraovarian injection of PRP. Of these women, 23 (7.4%) achieved spontaneous pregnancy, and another 82 (26.3%) developed at least one cleavage stage embryo after controlled ovarian hyperstimulation (COH) and IVF.

## RESULTS

**Figure 1:**
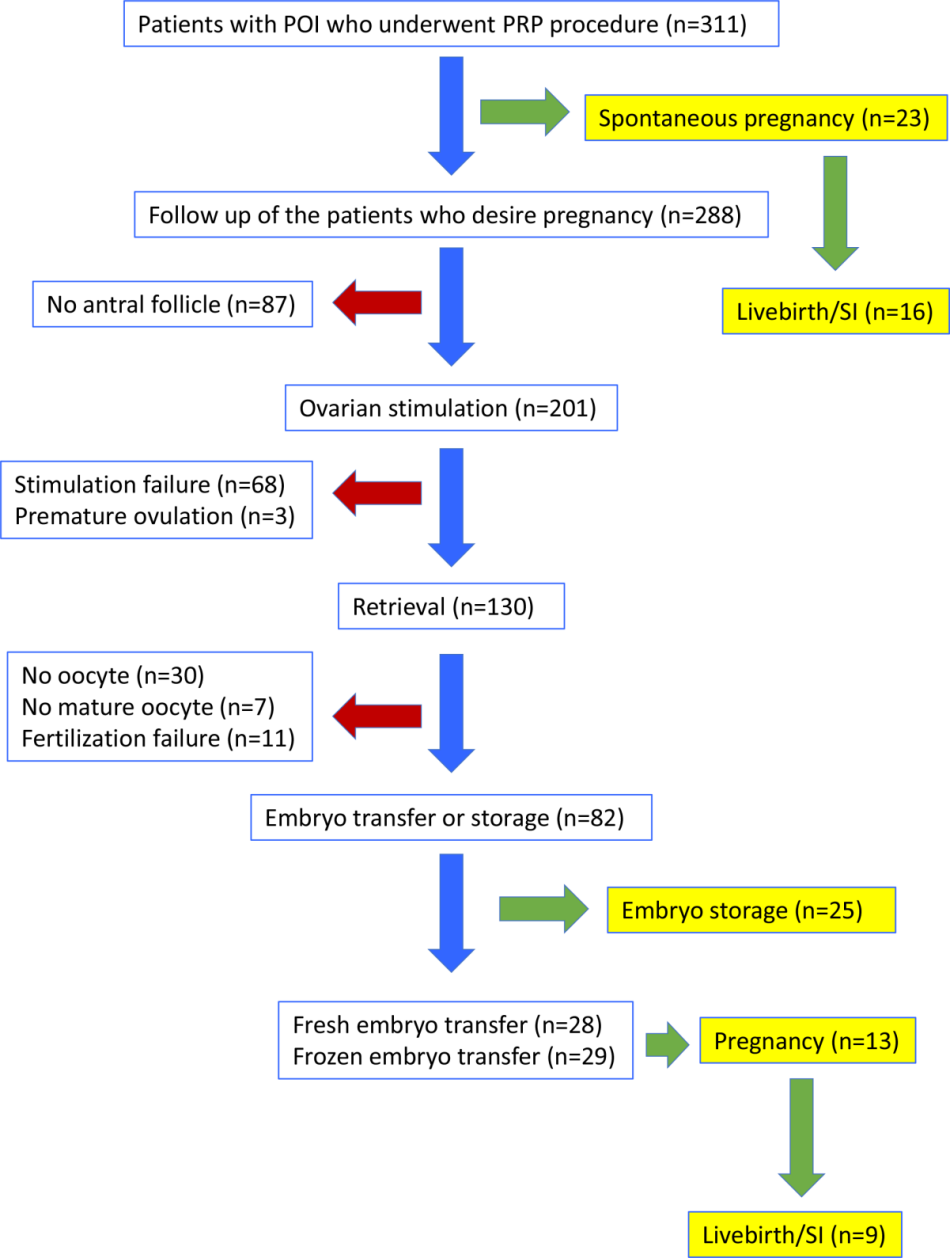
**Flow-chart of the clinical outcomes in women with POI who underwent PRP injection.** SI: sustained implantation.

A total of 311 women (mean age ± SD: 34.8 ± 4.3) with the diagnosis of POI were included in the study. Flowchart of outcomes is shown in Figure 1.

### Spontaneous pregnancies in response to PRP

Spontaneous pregnancy occurred in 23 women (7.4%, mean age ± SD: 34.6 ± 4.0) one or two cycles after the PRP procedure (Figure 1). Characteristics of these women are presented in Supplementary Table 1. At the time of this report, 7 of the spontaneously conceived pregnancies were lost as spontaneous miscarriages, while 5 were ongoing between 24^th^ to 35^th^ weeks of gestation and 11 were delivered between 37 to 40^th^ weeks of gestation. Therefore, 16/23 (69.5%) of spontaneous pregnancies that developed after PRP treatment resulted in sustained implantation or livebirth.

### Ovarian reserve assessment and initiation of controlled ovarian hyperstimulation

**Figure 2:**
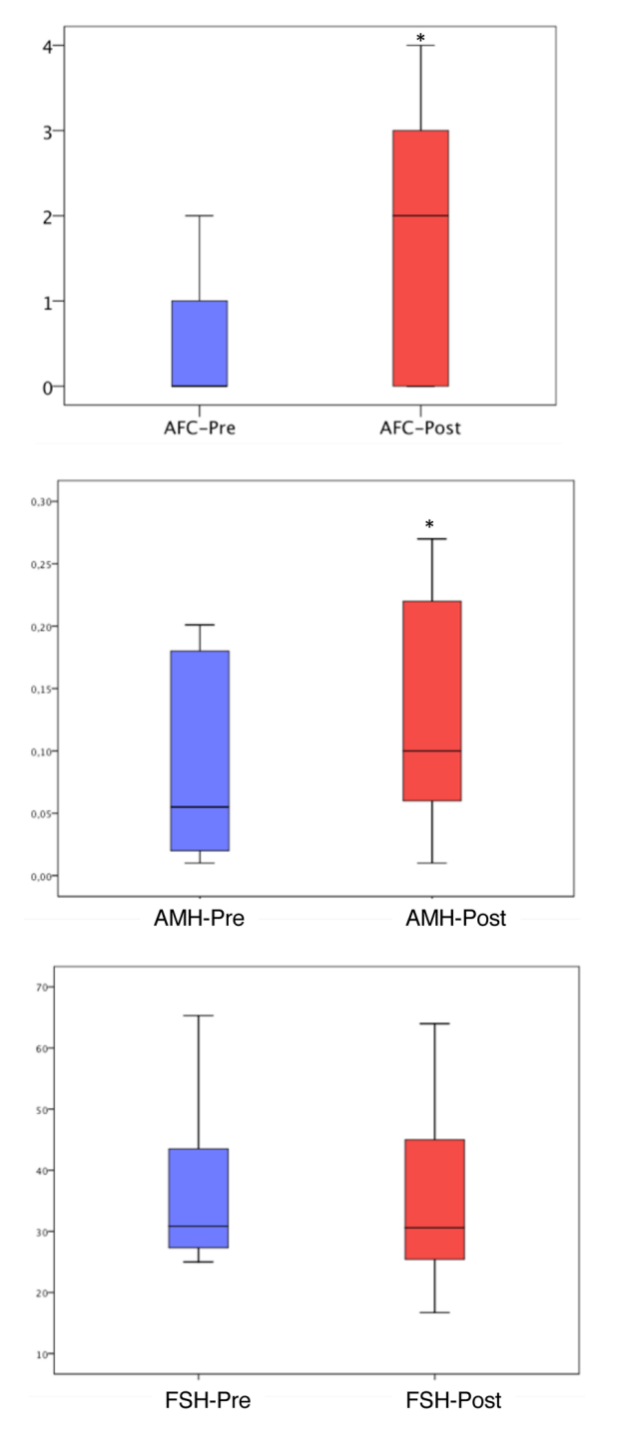
**Antral follicle count, AMH and FSH levels before and after PRP injection.** The ends of the boxes are the upper and lower quartiles, so the box spans the interquartile range (25^th^ to 75^th^ percentile). The horizontal line inside the boxplot represents the median value. The whiskers extend between 5%-95%. *: P<0.05

When ovarian reserve parameters were analyzed, we observed a statistically significant increase in antral follicle count following PRP treatment (1.7 ± 1.4 vs 0.5 ± 0.5; p<0.01). Notably, prior to the PRP treatment 186 of the 311 women had an AFC of “0”, while after PRP injection, only 87 had no antral follicles. Serum AMH also increased after PRP treatment (0.18 ± 0.18 vs 0.13 ± 0.16; p<0.01), while serum FSH was not statistically significantly different (41.6 ± 24.7 vs 41.9 ± 24.7; p=0.87) (Figure 2).

Due to spontaneous conception of 23 women following PRP treatment, only 288 were candidates for controlled ovarian hyperstimulation (COH). Among those, 201 (70%) developed antral follicles and underwent COH, while 87 did not (Figure 1). In 17 of the women without antral follicle after PRP injection, AMH levels increased and FSH levels decreased, however stimulation was not initiated due to inability to detect antral follicles over the 6 months of follow up.

Of the 201 women who underwent COH, 95 had no antral follicles prior to PRP and at least one follicle developed after PRP, another 94 had an increase in the number of existing antral follicles (minimum 1 and maximum 3), and 12 had the same number of antral follicles before and after PRP treatment. As per the study protocol, COH was not attempted in the first menstrual cycle after PRP injection. Among women who started COH, 22 (10.9%) started within the second menstrual cycle after PRP, 106 (52.7%) within the third cycle, 44 (21.9%) within the fourth cycle, 22 (10.9%) within the fifth cycle, and in 7 (3.5%) within the sixth cycle.

### IVF outcomes

IVF was attempted in 201 women who had at least one antral follicle after PRP (Figure 1, Table 1). Oocyte retrieval was performed in 130 (64.7% of stimulated) women, while 71 (35.3%) could not undergo oocyte retrieval due to stimulation failure (n=68) or premature ovulation (n=3). Among the couples in whom oocyte retrieval was performed, no oocyte was obtained in 30, mature oocytes could not be obtained in 7, and fertilization failure occurred in 11 women. In 82 women (40.8% of stimulated), at least one cleavage stage embryo was obtained and embryo cryopreservation or fresh embryo transfer was performed. These embryos were morphologically grade A/B. Mean number of oocytes per retrieval was 1.81 ± 1.30. The mean number of 2PN, and cleavage stage embryos obtained in women who developed embryos were, 1.24 ± 0.49, and 1.18 ± 0.39, respectively.

**Table 1:** Clinical and IVF outcome parameters of women with POI who underwent Intraovarian autologous PRP injection (Mean ± SD).

Patient age (all women, n=311)	34.8 ± 4.3
Partner Age (all men, n=311)	37.4 ± 5.6
Duration of infertility (years)	6.8 ± 4.9
Days of stimulation (in women who underwent ovarian stimulation; n=201)	8.6 ± 3.0
Total gonadotropin dose (IU) (in women who underwent controlled ovarian hyperstimulation; n=201)	5,156 ± 1,773
E2 level (pg/ml) on the day of hCG (in women who underwent retrieval; n=130)	231 ± 122
Number of retrieved oocytes (in women who had oocytes retrieved; n=100)	1.81 ± 1.30
Number of mature oocytes (in women who had mature oocytes retrieved; n=93)	1.47 ± 0.76
Number of 2 pronuclei embryos (in women with fertilization; n=82)	1.24 ± 0.49
Fertilization rate (%) (in women with fertilization; n=82)	55.8 ± 29.1
Number of cleavage stage embryos (in women with fertilization; n=82)	1.18 ± 0.39

Among the 82 women who developed embryos, 25 preferred to store cryopreserved embryos for transfer at a later stage, and 57 underwent embryo transfer. Of those who underwent embryo transfer, 28/57 (49.1%) were fresh embryo transfers and 29/57 (50.9%) were frozen-thawed embryo transfers; 7/28 (25.0%) of fresh embryo transfers and 6/29 (20.7%) of frozen-thawed embryo transfers resulted in a pregnancy. At the time of this report, among fresh embryo transfers, three were miscarried during the first trimester (42.9%), three were ongoing at 24, 30, and 31 weeks of gestation (42.9%), and one had delivered at 34 weeks of gestation (14.3%). When frozen embryo transfer pregnancies were evaluated, one was miscarried during the first trimester (16.7%), three were ongoing at 17, 22 and 36 weeks of gestation (50.0%), and two had delivered at 39 weeks of gestation (33.3%). In total, of the women who underwent embryo transfer after PRP treatment, 13/57 (22.8%) achieved pregnancy, and 9/57 (15.8%) achieved sustained implantation or livebirth.

### Baseline ovarian reserve parameters and response to treatment

We assessed AFC at baseline (prior to PRP injection) in association with the likelihood of producing at least one mature (metaphase 2, M2) oocyte or a cleavage stage embryo. We found that women with an AFC of 0 prior to treatment were less likely to have at least one M2 oocyte retrieved or cleavage stage embryo developed compared to those with an AFC of 1 or 2 (Tables 2, 3). Similar observations were made with baseline serum FSH and AMH levels (Tables 2, 3).

**Table 2:** The relationship between, pre-treatment measurements of ovarian response predictors and outcome (retrieval of a mature oocyte) after PRP treatment.

**AFC PRIOR TO PRP**	**<35 years old (N/Total (%))**	**35-37 years old (N/Total (%))**	**38-40 years old (N/Total (%))**	**All ages (N/Total (%))**
**0^a^**	11/73 (15.1%)*	9/37 (24.3%)	14/65 (21.5%)*	34/175 (19.4%)*
**1 and 2^b^**	19/34 (55.9%)*	10/25 (40.0%)	21/38 (55.3%)*	50/97 (51.5%)*
**AMH PRIOR TO PRP**	**<35 years old (N/Total (%))**	**35-37 years old (N/Total (%))**	**38-40 years old (N/Total (%))**	**All ages (N/Total (%))**
**<25^th^ percentile^c^ (0.01-0.02 ng/mL)**	2/34 (5.9%)^†^	4/15 (26.7%)	11/37 (29.7%)	17/86 (19.8%)^†^
**25^th^ -100^th^ percentile^d^ (0.03-0.82 ng/mL)**	28/73 (38.4%)^†^	15/47 (31.9%)	24/66 (36.4%)	67/186 (36.0%)^†^
**FSH PRIOR TO PRP**	**<35 years old (N/Total (%))**	**35-37 years old (N/Total (%))**	**38-40 years old (N/Total (%))**	**All ages (N/Total (%))**
**>75^th^percentile^e^ (44.2-155 mIU/mL)**	1/30 (3.3%)^‡^	2/12 (16.7%)	2/26 (7.7%)^‡^	5/68 (7.4%)^‡^
**0^th^ -75^th^ percentile^f^ (25-44.1 mIU/mL)**	29/77 (37.7%)^‡^	17/50 (34.0%)	33/77 (42.9%)^‡^	79/204 (38.7%)^‡^
**TOTAL**	30/107 (28.0%)	19/62 (30.6%)	35/103 (34.0%)	84/272 (30.9%)

Table shows the number of women who had at least one mature oocyte retrieved per women treated in each age group. Data shown as grouped according to pre-treatment AFC, AMH OR FSH. All women who participated in the study were included (n=311), except those who conceived spontaneously (n=23) and those who did not have all parameters recorded (n=16). *: p<0.05 in the age group according to pre-treatment AFC (0 vs 1 or 2); ^†^: p<0.05 in the age group according to pre-treatment AMH (<25^th^ percentile vs 25^th^-100^th^ percentile); ^‡^: p<0.05 in the age group according to pre-treatment FSH (>75^th^ percentile vs 0^th^-75^th^ percentile).

**Table 3:** The relationship between pre-treatment measurements of ovarian response predictors and outcome (obtaining a cleavage stage embryo) after PRP treatment.

**AFC PRIOR TO PRP**	**<35 years old (N/Total (%))**	**35-37 years old (N/Total (%))**	**38-40 years old (N/Total (%))**	**All ages (N/Total (%))**
**0**	9/73 (12.3%)*	9/37 (24.3%)	11/65 (16.9%)*	29/175 (16.6%)*
**1 and 2**	18/34 (52.9%)*	10/25 (40.0%)	20/38 (52.6%)*	48/97 (49.5%)*
**AMH PRIOR TO PRP**	**<35 years old (N/Total (%))**	**35-37 years old (N/Total (%))**	**38-40 years old (N/Total (%))**	**All ages (N/Total (%))**
**<25^th^ percentile (0.01-0.02 ng/mL)**	2/34 (5.9%)^†^	4/15 (26.7%)	9/37 (24.3%)	15/86 (17.4%)^†^
**25^th^ -100^th^ percentile (0.03-0.82 ng/mL)**	25/73 (34.2%)^†^	15/47 (31.9%)	22/66 (33.3%)	62/186 (33.3%)^†^
**FSH PRIOR TO PRP**	<35 years old (N/Total (%))	35-37 years old (N/Total (%))	38-40 years old (N/Total (%))	All ages (N/Total (%))
**>75^th^percentile (44.2-155 mIU/mL)**	1/30 (3.3%)^‡^	2/12 (16.7%)	2/26 (7.7%)^‡^	5/68 (7.4%)^‡^
**0^th^ -75^th^ percentile (25-44.1 mIU/mL)**	26/77 (33.8%)^‡^	17/50 (34.0%)	29/77 (37.7%)^‡^	72/204 (35.3%)^‡^
**TOTAL**	27/107 (25.2%)	19/62 (30.6%)	31/103 (30.1%)	77/272 (28.3%)

Table shows the number of women who had at least one mature oocyte retrieved per women treated in each age group. Data shown as grouped according to pre-treatment AFC, AMH OR FSH. All women who participated in the study were included (n=311), except those who conceived spontaneously (n=23) and those who did not have all parameters recorded (n=16). *: p<0.05 in the age group according to pre-treatment AFC (0 vs 1 or 2); †: p<0.05 in the age group according to pre-treatment AMH (<25^th^ percentile vs 25^th^-100^th^ percentile); ‡: p<0.05 in the age group according to pre-treatment FSH (>75^th^ percentile vs 0^th^-75^th^ percentile).

## DISCUSSION

In this study, we investigated whether intraovarian injection of autologous PRP would improve ovarian reserve parameters, ovarian response to stimulation, and result in live births in women diagnosed with POI. Intraovarian PRP injection was performed in a total of 311 women. Among them, 25 women (8.0%) achieved livebirth/sustained implantation (either spontaneously or after IVF), while another 25 (8.0%) stored cryopreserved embryos.

PRP has been utilized and studied since 1970s and has been implemented into routine clinical practice as a rejuvenating agent or to promote healing in dermatology, plastic surgery, dentistry, and orthopedics [17]. Similarly, in obstetrics and gynecology, several studies with small sample size were conducted to investigate the effects of PRP injection into the uterus and ovaries [18]. The first data about intraovarian injection of PRP were published by Sills et al. [19]. They reported improvement in the laboratory parameters after intraovarian PRP in four women with DOR. Subsequently, Sfakianoudis et al. reported the first pregnancy in a menopausal woman after intraovarian PRP injection [20]. This was a 40-year-old woman diagnosed with POI at the age of 35. Her serum biomarkers of ovarian reserve improved after the PRP procedure and pregnancy was achieved after natural IVF; however, miscarriage occurred in the fifth week of gestation. Examining an in vitro model, Hosseini et al. reported a significant increase in the development of early antral follicles after they were cultured with PRP, concluding that it may be an effective method to induce follicular development [16]. Our study builds on these previous reports and using large sample size, provides data that can guide future research and help patient counseling.

In women with the diagnosis of POI, extremely low ovarian reserve parameters are not always associated with absence of follicles in the ovary. Kawamura et al. removed ovarian tissue from 27 women with POI through laparoscopic surgery and cut these tissues into strips [8]. After Hippo signaling disruption and Akt stimulation, small fragmented ovarian strips were autotransplanted beneath the serosa of Fallopian tubes. Follicular growth was observed in 8 of these 27 women (29.6%), all of which developed preovulatory follicles within less than 6 months. Retrieval of mature oocytes occurred in 5 women (18.5% of total), resulting in a livebirth after embryo transfer. In a follow up study from the same group, 37 women with POI were treated similarly, resulting in retrieval of mature oocytes from 6 women (16%), and 2 livebirths (5.4%) [9]. More recently, Kawamura et al. have described a drug-free approach based on Hippo signaling disruption alone by performing partial ovarian cortical removal in women with DOR and autografting back during the same laparoscopic surgery [21]. They treated 11 women with severely diminished ovarian reserve, 9 had an increase in AFC and underwent IVF-ICSI. In addition to one spontaneous pregnancy, embryo transfer resulted in one live birth, and two ongoing pregnancies. Three additional women had embryos cryopreserved for future transfer. In our study, 23 women conceived spontaneously (7.4%), and antral follicle development was observed in 70% of the rest of the study group (201/288). Of these 201 women, 82 generated embryos; 57 underwent embryo transfer and 25 of them cryopreserved embryos to be transferred at a later stage. Therefore, autologous PRP injection seems to result in encouraging outcomes, without the complexity of ovarian surgery.

Spontaneous pregnancy is uncommon in women with POI, with reports of spontaneous conception ranging from 2.2% to 14.2% [22]. It is noteworthy that none of the women in our study group had a history of spontaneous pregnancy before PRP, and the 23 spontaneous pregnancies that occurred after PRP, all happened within the first two cycles after the procedure. Similarly, Pantos et al. recently reported two women with POI aged 27 and 40 years, and one postmenopausal women aged 46 years to conceive spontaneously after PRP injection [23]. Spontaneous pregnancy was also reported by Herraiz et al. in 3 of 17 women with DOR who received autologous bone marrow derived stem cell injection into their ovarian artery [10]. We found that 7.4% of women with POI treated with PRP spontaneously conceived. The mechanisms by which ovarian activation strategies induce spontaneous conception are worthy of further investigation.

PRP contains a number of active substances [11]. However, PRP‘s mechanisms of action in general, and its effects in the ovary in particular, remain largely unknown. Within this context, our study provides relevant information. First, among women who developed at least one antral follicle after PRP injection and were therefore able to undergo IVF in our study, 64% started within the first three cycles and another 21% in the fourth cycle. The progression of human follicles from a primary follicle to ovulation is estimated to require approximately 85 days [24]. This development occurs in gonadotropin independent and dependent phases [25]. Our findings suggest that any type of follicle present in the ovary may be directly affected by PRP. In addition, indirect effects through action on somatic compartment are also possible. Second, we found that women who did not have an antral follicle at the time of PRP injection were less likely to respond to treatment compared to those who had one or two antral follicles (Tables 2, 3). Similarly, women in the lowest quartile for serum AMH and highest for serum FSH, were less likely to respond (Tables 2, 3). Collectively, our findings suggest that PRP helps activate existing preantral and/or early antral follicles, and that the number of remaining follicles in the ovaries of women with POI likely determines the extent of their response. PRP treatment resulted in unchanged levels of serum FSH, with minimal improvement in AMH and AFC (Figure 2), supporting this conclusion.

Among 201 women in whom COH was attempted in this study, 82 (40.8%) had at least one cleavage embryo developed. While this is a very encouraging result, when 55 of these 82 women underwent embryo transfer, only 9/55 (16.3%) achieved sustained implantation and livebirth. This attrition could be due to a number of factors. First, at the center where the study is conducted, embryos of women with POI are often transferred or cryopreserved at the clevage stage, associated with a lower implantation rate compared to blastocysts. Second, preimplantation genetic testing for aneuploidy (PGT-A) was not used, therefore aneuploid embryos were not excluded from transfers. Finally, it is possible that activation of otherwise dormant follicles in women with POI might recruit oocytes with lower viability.

This study has a number of limitations. First, the pregnancy rates after embryo transfer could not be conclusively established as some of the women conceived spontaneously and others opted to store cryopreserved embryos. Second, in this study we compared patients’ outcomes to their pre-treatment state. Therefore, our study does not include an independent control group and is not randomized. The description of a control group is also open for debate. Based on the work by Kawamura et al. [8, 21], one might ask whether the observed effect of PRP could be primarily mechanical. In the same context, Zhang et al. have evaluated follicle development and pregnancy outcome in 80 women with POI after ovarian biopsy/scratch [26]. They reported that they obtained mature oocytes in 10 women (12.5%) and one (1.25%) delivered a healthy singleton baby after IVF. At present, there are no existing data on sham/vehicle injections as controls. Clinical delivery of cells or cell products to the ovary (directly or via the ovarian artery) may be having their effects due to the injection, or, due to vehicle effects, and not biomolecules or cells. Until specific molecules that have reproducible effects are identified, such a mechanism for PRP effect should be considered as a possibility [26].

In conclusion, in women with POI, intraovarian injection of autologous PRP might be considered as an alternative experimental treatment option. Future studies with larger sample size and randomized prospective study design are necessary to determine whether this intervention truly results in improved clinical outcomes, through spontaneous conception and/or assisted reproduction. Until then, autologous PRP treatment should not be recommended as part of routine treatment in women with POI.

## MATERIALS AND METHODS

### Patient selection

Women diagnosed with POI based on European Society of Human Reproduction and Embryology (ESHRE) criteria ((i) oligo/amenorrhea for at least 4 months, (ii) an elevated serum FSH >25 IU/l on two occasions 4 weeks apart (iii) onset before the age of 40 years) [5] and treated with intraovarian PRP injection at Acibadem Maslak Hospital, in Istanbul, Turkey, between November 2018 and October 2019, were included in the study. Inclusion criteria were < 40 years of age, history of infertility for > 1 year, and having at least one ovary. Exclusion criteria were history of malignancy, ovarian insufficiency of genetic etiology, prior major lower abdominal surgery resulting in pelvic adhesions, anticoagulant use for which plasma infusion is contraindicated, and current or previous IgA deficiency. The study protocol was approved by the University’s institutional review board and ethics committee (ATADEK-2019/8-18). All women included in the study signed a consent form.

### PRP preparation

PRP preparation was performed by separating platelet-rich plasma after centrifugation. Approximately 20 ml of blood sample was collected under sterile conditions, and PRP was prepared using T-lab autologous platelet-rich plasma kit (T-Biotechnology Laboratory, Bursa, Turkey) according to the manufacturer’s instructions. Briefly, after blood collection, the tubes were centrifuged at 830 g for 8 minutes. Then, a 16 G needle connected to a 5 ml syringe was inserted into the tube and advanced to the buffy coat layer. The PRP was collected by rotating the needle tip. After collecting approximately 2-4 cc PRP from the first tube, the second tube was processed similarly (a total 4-8 cc PRP was collected). The collected solution was transferred to the re-suspension tube and shaken gently for 30s-1 min.

### Intraovarian injection

The same day, within 2 hours of sample preparation, PRP injection was performed transvaginally under ultrasound guidance and under sedation anesthesia into at least one ovary using a 35 cm 17 G single lumen needle. The injection was done underneath the ovarian cortex to the subcortical and stromal areas. Although women with POI have small fibrotic ovaries making intraovarian injection of 2-4 ml of PRP challenging, this was achieved through distention, possibly by creation of new planes and making the injection in multiple sites within the ovaries. After the procedure, the patients were taken to the recovery room and observed for 30-40 minutes and discharged home on the same day.

### Timing of PRP injection, patient assessment and follow-up

PRP injection was timed randomly in women who were amenorrheic, while in women who reported oligomenorrhea, PRP was injected within 10 days after completion of menstrual bleeding. Baseline antral follicle count (AFC), serum anti-mullerian hormone (AMH) and follicle stimulating hormone (FSH) levels were determined prior to PRP injection, on the same day. Intra-assay coefficient of variation for AMH was <10%, inter-assay coefficient of variation was <12%, detection range was 0.156–10 ng/mL, and minimum detectable concentration was < 0.055 ng/mL. Intra-assay coefficient of variation for FSH was <5.1%, inter- assay coefficient of variation was <7.6%, and minimum detectable concentration was 0.22 mIU/ml.

After PRP injection, both amenorrheic and oligomeorrheic women were managed expectantly for 6 weeks to allow spontaneous pregnancy or menses. Those who did not have a menstrual bleed for 6 weeks underwent pregnancy testing and (if not pregnant) menses was hormonally induced (2x (2 mg estradiol valerate) for 5 days and 2x (2 mg estradiol valerate + 0.5 mg norgestrel) for 5 days (Cycloprogynova, Bayer)). The same strategy was repeated if menstruation was delayed in the subsequent cycle. On the 2-4^th^ days of the second menstrual cycle after the PRP procedure, AFC and serum AMH and FSH levels were re-assessed. Those who were found to have antral follicle(s) at that point were started on controlled ovarian hyperstimulation (COH), while those who did not were followed monthly, up to 6 months, and underwent COH when/if they developed antral follicle(s). Following each assessment, patients who did not develop antral follicles were treated with cyclic estrogen and progesterone (2 mg estradiol valerate for 10 days and 2 mg estradiol valerate + 0.5 mg norgestrel for 11 days (Cycloprogynova, Bayer)). This was done for ease of scheduling and to control cycle progress.

Positive response to PRP was considered when at least one antral follicle was seen on ultrasound in women without any antral follicles at baseline or an increase in AFC compared to baseline measurements or an increase in AMH or a decrease in serum FSH levels in women with the same AFC when compared to pre-treatment measurement.

Women who were found to have at least one antral follicle (even if the number was not higher compared to pre-PRP evaluation) were started on ovarian stimulation for IVF-ICSI. In cases with an increase in AMH or decrease in FSH, but no antral follicles compared to basal measurements, stimulation was not started.

### Controlled ovarian hyperstimulation and IVF

Controlled ovarian hyperstimulation (COH) was started on the second or third day of the induced menstrual cycle. Gonadotropin stimulation was started at 300 IU recombinant FSH (randomized randomly Gonal F; Merck, or Fostimon; IBSA) and 300 IU human menopausal gonadotrophin (hMG) (Merional; IBSA). When the dominant follicle reached a mean diameter of 14 mm, cetrorelix 0.25 mg /d s.c (Cetrotide; Merck) was administered. Patients were monitored with serial serum E2 and progesterone measurements and transvaginal ultrasonographic examinations. When at least one leading follicle reached a mean diameter of 18 mm, 250 mcg recombinant chorigonadotrophin alfa (rHCG, Ovitrelle; Serono) was used to induce follicle maturation.

Oocyte retrieval was carried out under transvaginal ultrasound-guided puncture 34 h after rhCG administration. Oocyte denudation was done 4 hours after retrieval and ICSI was performed to all women. Good quality embryos were frozen at cleavage stage for embryo cryopreservation, or Day 3 cleavage stage or day 5 blastocyst embryos were transferred under ultrasound guidance, based on patient and physician preference.

For the women with frozen-thawed embryo transfer protocol, oral contraceptives were started on the 2-5^th^ of the preceding menstrual cycle followed by sc injection of 3.75 mg leuprolide acetate depot (Lucrin; Abbott) in the midluteal phase. Endometrial priming was started on the second day of next menstrual cycle after making sure that an early proliferative endometrial pattern was confirmed on transvaginal ultrasound and E2 and P levels were suppressed. Estradiol (2 mg Estrofem; Novo Nordisk) was orally administered at a dose of 4mg for 5 days. The dose was increased sequentially to 6 mg for 4 more days and 8 mg for 5 more days. At the end of 14 days of estradiol use, transvaginal ultrasound evaluation of the endometrium was monitored with E2 and P level assessments. If the endometrial thickness was over 8 mm and P level was below 1.5 ng/ml, then vaginal progesterone (Crinone gel 8% BID; Merck) was started twice a day and frozen-thawed embryo transfer was scheduled. If the endometrial thickness was below 8 mm, estradiol patch 7.8 mg (Climara; Bayer) was administered via the transdermal route and checked again after 4 days later. If the thickness was still below 8 mm, then the cycle was cancelled.

In women undergoing fresh embryo transfer, daily vaginal progesterone (Crinone gel 8% BID; Merck) was used for luteal phase support, starting on the day after oocyte retrieval. Pregnancy outcome was determined by assessing serum ß-HCG level 12- 14 days after embryo transfer.

Clinical pregnancy was defined as a pregnancy that is confirmed by both high levels of hCG and ultrasound confirmation of a gestational sac or fetal pole. Sustained implantation was defined as women who were discharged from care at 12 weeks of pregnancy with a fetal heartbeat.

### Statistical analysis

Data distribution was tested with the Shapiro–Wilk test. Differences between pre- and post-treatment values were compared using the Wilcoxon test. Logistic regression was used to estimate the odds ratio of having at least one mature oocyte or at least one cleavage stage embryo in percentile categories after adjusting for age. Stratified analysis was also performed to investigate the odds ratio among women in different age groups. As post-PRP outcomes have not yet been determined in cohort studies of adequate samples size, we were not able to perform a reliable power analysis prior to the initiation of the study. All data were analyzed using SPSS (SPSS-IBM 2.3, Inc., Chicago, IL, USA). p values less than 0.05 were considered statistically significant.

## Supplementary Material

Supplementary Table 1
