# Supplementary material for: Effects of intraovarian injection of autologous platelet rich plasma on ovarian reserve and IVF outcome parameters in women with primary ovarian insufficiency
Source: Aging (Albany NY). 2020 Jun 5;12(11):10211–22. doi: 10.18632/aging.103403 (PMC7346073; doi:10.18632/aging.103403)
Supplement: Supplementary Table 1 [file aging-12-103403-s001..pdf]

SUPPLEMENTARY TABLE

Supplementary Table 1. Characteristics of 23 women who had spontaneous pregnancy following PRP injection.

	**Mean ± SD**
Age	34.6 ± 4.0
Duration of infertility	5.6 ± 3.4
Prior IVF attempts	2.6 ± 2.1
FSH (mIU/mL) prior to PRP	33.3 ± 8.9
AMH (ng/ml) prior to PRP	0.09 ± 0.07
AFC prior to PRP	1.26 ± 0.8
